# Editorial: Extracellular RNAs as Outside Regulators of Gene Expression in Homeostasis and Pathology

**DOI:** 10.3389/fcell.2021.818430

**Published:** 2022-01-06

**Authors:** Tatiana Lopatina, Darius Widera, Anastasia Efimenko

**Affiliations:** ^1^ Department of Medical Sciences, University of Turin, Turin, Italy; ^2^ Stem Cell Biology and Regenerative Medicine Group, School of Pharmacy, University of Reading, Reading, United Kingdom; ^3^ Institute for Regenerative Medicine, Medical Research and Education Center, Lomonosov Moscow State University, Moscow, Russia

**Keywords:** extracellular RNA, extracellular vesicles, noncoding RNA, microRNA, cell-to-cell communication, biomarkers

The classic view of ribonucleic acids (RNAs) puts emphasis on their central involvement in providing the genetic information for the protein synthesis. However, recent findings have indicated that RNAs can shuttle between various cells of one organism and could be transported also between different organisms (Xiang et al., 2006). Membrane vesicles or protein complexes protect extracellular RNAs (exRNAs) in extracellular space and within biological liquids such as plasma, milk, or cerebrospinal liquid. Since Mandel and Metais first discovered extracellular nucleic acids in human plasma in 1948, exRNA was believed to be a waste material released by damaged cells. Only after discovery of RNA interference, extracellular microRNAs (miRNAs), long non-coding RNAs (lncRNAs), and other regulatory types of RNA in the 2000s, study of regulatory functions of RNAs caught the attention of the field. During the two last decades, it has been shown that exRNAs are not only specific biomarkers for different diseases and conditions but also active players in the regulation of inflammation, cell activation, apoptosis, and migration.

This Research Topic has invited original research articles and reviews focused on all aspects of exRNA biology including but not limited to the role of exRNAs in cell-cell communication, their potential as biomarkers, and their therapeutic potential. The topic collected five original research articles and three reviews.

In the study by Vulf et al., several microRNAs (miRNAs) such as let-7c-5p, miR-15b-5p, miR-21-5p, miR-423-5p, and miR-143-3p have been revealed as novel potential biomarkers of non-alcoholic fatty liver disease. The authors demonstrated that these circulating miRNAs are differentially expressed in serum derived from patients with steatosis and steatohepatitis and are involved in the regulation of genes relevant to the insulin resistance and development of non-alcoholic fatty liver disease.

In another study within this Research Topic, Cai et al. eloquently demonstrated the advantages of using circulating exRNAs as potential biomarkers and therapeutic targets for Parkinson’s disease (PD). The authors identified novel aberrantly expressed microRNAs in both the plasma and extracellular vesicles (EVs) of PD patients, validated them in clinical samples, as well as in cell and animal models of PD. They also characterized the complicated regulatory network involved in miRNA-mediated modulation of targets genes related to PD pathogenesis. In particular, miR-23b-3p was identified as a novel direct regulator of alpha-synuclein, a crucial pathogenic hallmark of PD.

The secretion of exRNA within EVs is a highly regulated process that depends on the cells of origin, their metabolism, activation status, senescence, age, and differentiation. This is highly relevant for EV function and explains contradictions in the literature about pro- and anti-inflammatory characteristics of mesenchymal stem cell-derived EVs.

In the comprehensive review by Cai et al., the role of different classes of non-coding RNAs (ncRNAs), including miRNAs, long non-coding ncRNAs, and circulating RNAs (circRNAs) in cellular senescence are discussed. In this review, exRNAs have been recognized as essential components of the senescence-associated secretory phenotype that altered cells acquire during senescence. Moreover, they postulated that exRNAs as EV cargo can affect the behavior of neighboring cells via autocrine/paracrine mechanisms leading to reprogramming of the microenvironment toward a pro-senescent state. The authors focused on MSC senescence and demonstrated that exRNAs could regulate MSC functions during the aging of an organism contributing to the progressive decrease in tissue maintenance and regeneration. Furthermore, they highlighted the impact of senescence on MSC-derived therapeutic products such as MSC-EVs and suggested strategies targeting MSCs and specific ncRNAs.

Manipulation of parental cells for EV engineering could non-specifically change the molecular composition of EVs, as demonstrated by several research groups (Lombardo et al., 2018; Lopatina et al., 2018). Zubkova and colleagues showed that virus transduction of mesenchymal stem cells changed the pattern of microRNA within EVs independently from the transgene (green fluorescent protein or stem cell factor) (Zubkova et al.). Cell transduction with adeno-associated virus is of high interest since this virus is used clinically and could represent an effective tool for engineering EV with a desired molecular cargo. Nevertheless, stress from the transduction had collateral effects on the miRNA cargo of MSC-derived EVs. Overall, microenvironmental stress leads to substantial differences in the miRNome of the EVs.

Release of exRNA affects both the target and parental cells. The decreased concentration of miRNAs in the parental cells as a result of their secretion affects the post-transcriptional gene repression within these cells. Di Silvestre et al. showed that activation of T cells led to an active release of miRNAs (miR-21-5p, miR-155-5p, miR-150-5p, miR-142-3p, and miR-106a-5p), inhibited expression of pro-inflammatory genes, thus permitting proliferation and acquisition of T cell effector phenotype. On the other hand, EVs from these activated T cells could control overall inflammation and induce T regulatory cell formation in the target recipient cells by their inhibitory miRNAs.

Several distinguished features make EVs suitable and desirable as therapeutic platforms for targeting of many pathological conditions with exRNAs as key therapeutic agents. A study by Chiabotto et al. illustrates this approach with regards to the novel treatment option for liver fibrosis. In this study, EVs derived from human liver stem cells (HLSC-EVs) were used. The authors demonstrated that specific microRNAs within HLSC-EVs, particularly miR-146a-5p, can revert the activated phenotype of hepatic stellate cells and thus counteract the development of hepatic fibrosis.

The role of exRNAs in the regulation of immune response gathers considerable interest in light of their involvement of chronic inflammation in numerous diseases. Yusof et al. described regulation of the lymphedema by immune-relevant exRNAs. Most of the described miRNAs (miR-155, miR-146, miR-92a, miR-31, and miR-21), were also investigated in other pathological conditions since they played a general inflammatory role (Sheedy and Frederick, 2015; Lee et al., 2016; Mahesh and Biswas, 2019; Wang W.-Y. et al., 2019; Wang Y. et al., 2019).

In addition to their action in the context of an organism, exRNAs can perform biological functions in the recipient organism. In their article, Jiang et al. reviewed summarised information on miRNAs present in breast milk-derived EVs. Based on *in vitro* and *in vivo* data from various research groups, they concluded that miRNAs from breast milk exhibit pro-regenerative and proliferative action on intestinal cells, anti-inflammatory effect on immune cells, and protect from oxidative stress (Jiang et al.). They highlight that the most abundant miRNAs were pluripotency-related miRNAs such as miR-148, let-7, miR-30b, miR-182, miR-200, and the miR-17-92 cluster. Interestingly, some of these miRNAs were detected even in neural cells after ingestion of milk EVs. Notably, the pattern of milk exRNA is different during the lactation period and is also affected by the health status of the mother. In this context, it has been shown that maternal nutrition, disease, lifestyle, or its stress result in changes of the expression of miRNAs relevant for immune regulation.

Given their fundamental involvement in regulation of basal functions of cells, organs, and organisms, it can be assumed that exRNAs can be exploited as a powerful tool for the regulation of all biological processes within one organism. Moreover, exRNAs have the potential to influence recipient organisms after transplantation or even ingestion. This makes exRNAs promising biomarkers and therapeutic tools that can be employed in a magnitude of diseases and conditions. However, evidence-based research and rigor should dictate translation of exRNA research into the clinic. Thus, a defined mode of action needs to be identified prior to clinical application of exRNAs. In addition, potential toxicity needs to be excluded and superiority over a placebo in an appropriate animal model demonstrated even in case of compassionate use of exRNAs in patients. In the view of gene therapy, RNA vaccines, and epigenetic gene expression regulation, investigations of the exRNA will give great insights for the possible therapeutic applications.
